# Radiotherapy in practice radioisotope therapy

**DOI:** 10.1038/sj.bjc.6603858

**Published:** 2007-07-31

**Authors:** E Glatstein

**Affiliations:** 1 University of Pennsylvania Medical Center, Philadelphia, PA, USA

This is a relatively short paperback book that the editor hopes will ‘overcome many of these obstacles (under utilization and limited accessibility) with contributions from experts in the field identifying the current role of radioisotope therapy in modern medicine and also providing a background in the physics and developmental biology that underpins their use’.

It succeeds to some degree. The authors are all British and they know their field well. They do discuss the role of radioisotope therapy for a variety of conditions and the book does provide reasonably well much on the physics of radioisotope therapy. In asmuch as each of the chapters is written by a different contributor(s), there is some overlap and there is an unfortunate tendency to use acronyms and abbreviations, sometimes without defining them, at least in some of the chapters. This book will give a good review for someone who is studying for his board examination. However, if one is in the early part of his/her career or has very limited background of isotope therapy, this book will not be as easy to learn from as others. The chapters are well written, but most of them do not have a great deal of detail. The exceptions to this are the chapters on antibody-directed radioisotope therapy by Dearling and Pedley, and the chapter specifically on radioimmunotherapy for lymphoma by Illidge and Du. Both these chapters are filled with detail and excellent references. The final chapter on radioprotection and regulatory aspects of radioisotope therapy is certainly good from the UK/European point of view; there is a small section towards the end on non-UK legislation, but it is actually pretty brief. Also, I would have appreciated it if this chapter on radioprotection would have had a few pages on how to potentially deal with, at the hospital-based level, mass casualties from radiation bio-terrorism. A nuclear explosion obviously would have major consequences on the basis of the explosion itself, but much of the patient damage would be owing to secondary radioisotope contamination. The so-called ‘dirty bomb’ would be predominately isotope contamination as the source of patient injury and chaos. Something on that topic would have been appropriate for this monograph. Finally, the index is suboptimal.

Keeping such criticisms aside, this is a well-written monograph and it should be an excellent review for someone studying for boards. It can also serve as a useful reference for someone who is not actively carrying out such treatments but who is nonetheless interested in the topic. For a nuclear medical specialist, this book would be insufficient.

